# Stroke risk in hypertrophic cardiomyopathy patients with atrial fibrillation: a nationwide database study

**DOI:** 10.18632/aging.104133

**Published:** 2020-11-23

**Authors:** Jung-Chi Hsu, Ya-Ting Huang, Lian-Yu Lin

**Affiliations:** 1 Division of Cardiology, Department of Internal Medicine, Camillian Saint Mary’s Hospital Luodong, Yilan, Taiwan; 2 Department of Nursing, Camillian Saint Mary’s Hospital Luodong, Yilan, Taiwan; 3 Division of Cardiology, Department of Internal Medicine, National Taiwan University Hospital, Taipei, Taiwan; 4 Internal Medicine, College of Medicine, National Taiwan University, Taipei, Taiwan; 5 Electrophysiology, Cardiovascular Center, National Taiwan University Hospital, Taipei, Taiwan

**Keywords:** hypertrophic cardiomyopathy, atrial fibrillation, stroke, transient ischemic attack, age

## Abstract

Current treatment guidelines recommend anticoagulation for hypertrophic cardiomyopathy (HCM) with atrial fibrillation (AF) regardless of the CHA_2_DS_2_-VASc score. As aging and stroke risk factors (hypertension, diabetes mellitus) are confounders of ischemic stroke, young patients with a low stroke risk may not need anticoagulant treatment. This study aimed to determine the incidence of stroke and its risk factors in HCM patients with AF during a long-term follow-up. Using a national database, we retrospectively investigated 18,724 HCM patients from a systematic sample of 1,000,000 Taiwanese people between 1997 and 2013. The incidences of AF and stroke were estimated. Data were analyzed using Cox regression models. AF was identified in 598 patients (262 men, mean age 66.3±13.0 years) during a median follow-up of 7.0 years. The AF incidence in HCM patients was 5.83 per 1000 person-years, and the overall incidence of AF-associated stroke was 24.14 per 1000 person-years. The incidence of transient ischemic attack (TIA)/ischemic stroke varied from 20.41 to 60.55 per 1000 person-years, without proportionality to CHA_2_DS_2_-VASc score increase. Among patients aged <40 years, none experienced TIA/ischemic stroke. Univariate Cox regression models showed that age (p<0.001), prior TIA/ischemic stroke (p=0.02), and CHA_2_DS_2_-VASc score (p=0.003) were risk factors for TIA/ischemic stroke. Multivariate analysis indicated that age (hazard ratio 1.04, 95% confidence interval [CI] 1.02-1.06, p=0.001) and prior TIA/ischemic stroke (hazard ratio 2.82, 95% CI 1.27-6.25, p=0.011) were independently associated with TIA/ischemic stroke. Taiwanese patients with concomitant HCM and AF have a high stroke risk regardless of the CHA_2_DS_2_-VASc score. Aging is the main predictor. As the overall incidence of stroke was low in young patients, anticoagulants may not be needed in this subpopulation.

## INTRODUCTION

Hypertrophic cardiomyopathy (HCM) is a common genetic cardiac disorder with an autosomal dominant mechanism of inheritance and a prevalence of 1:500 in adults worldwide. HCM is associated with an increased risk of left ventricular outflow tract obstruction, ventricular dysfunction, atrial and ventricular arrhythmias, and sudden cardiac death [1]. Atrial fibrillation (AF) is the most common arrhythmia in HCM; however, the outcomes of ischemic attack and stroke have been seldom studied and previously underestimated. This is a key issue in preventing thromboembolic events in HCM patients with AF.

The prevalence of AF in patients with HCM has ranged from 22% to 32% depending on the severity of HCM Overall, the incidence is about 2% per year [1]. Among the thromboembolic events in HCM, AF is frequently documented to be the underlying etiology. Either paroxysmal or persistent AF was found to increase the risk of stroke and peripheral vascular events by five times [2]. A previous study estimated that about 10% of HCM patients with a zero CHA_2_DS_2_-VASc score experienced thromboembolic events within 10 years, for which age, AF, prior thromboembolism, New York Heart Association class, and left atrial diameter were identified as independent predictors [3].

According to the database of the National Health Insurance Service in Korea, the prevalence of AF in HCM patients in Asia increased from 13.4% in 2005 to 20.9% in 2015, and the overall incidence rate of AF-associated stroke was 2.94 per 100 person-years [4]. The prevalence of stroke in HCM patients with AF was about 20% in Korea, which was much higher than that reported in other cohort studies in patients without HCM. This implies that Asian people with HCM might have higher risks of stroke when they develop AF. This hypothesis has been proven in a cohort study in Japanese patients with nonvalvular AF (NVAF) and a low CHA_2_DS_2_-VASc score of 0 or 1. In that study, HCM patients had a significantly higher incidence of thromboembolism than non-HCM patients (5.9% vs. 0.9%) [5]. Furthermore, another Korean study in patients with NVAF also revealed that the risk of stroke in patients with NVAF with HCM without any CHA_2_DS_2_-VASc stroke risk factors was similar to that in non-HCM patients with a CHA_2_DS_2_-VASc score of 3 [6]. All these studies indirectly support the current European Society of Cardiology guidelines recommending anticoagulation therapy for all HCM patients with AF regardless of the CHA_2_DS_2_-VASc score, and suggest considering HCM as an independent entity [5, 7, 8].

Although HCM patients with AF have a higher risk of stroke, not all of them have a stroke risk that requires anticoagulation therapy. For example, a 20-year-old HCM patient with AF may not need anticoagulation therapy. In this study, we aimed to investigate the stroke risks in a group of HCM patients with AF with a variety of background risk factors by using a systematic sample from a national cohort. We intended to determine the characteristics of patients who experienced a stroke during a long follow-up period. This might help guide the delivery of anticoagulation therapy precisely to patients with a high risk of stroke.

## RESULTS

**Figure 1:**
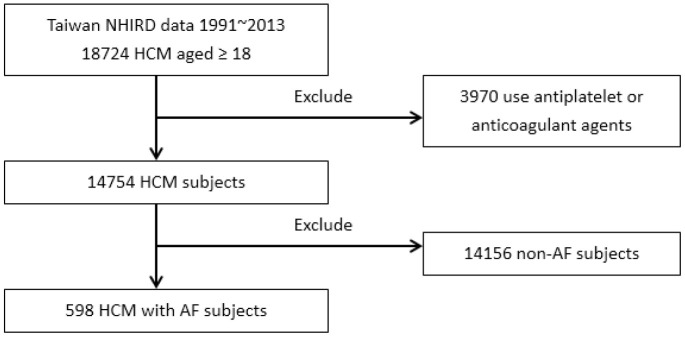
**Enrollment of patients with HCM and AF.**

**Figure 2:**
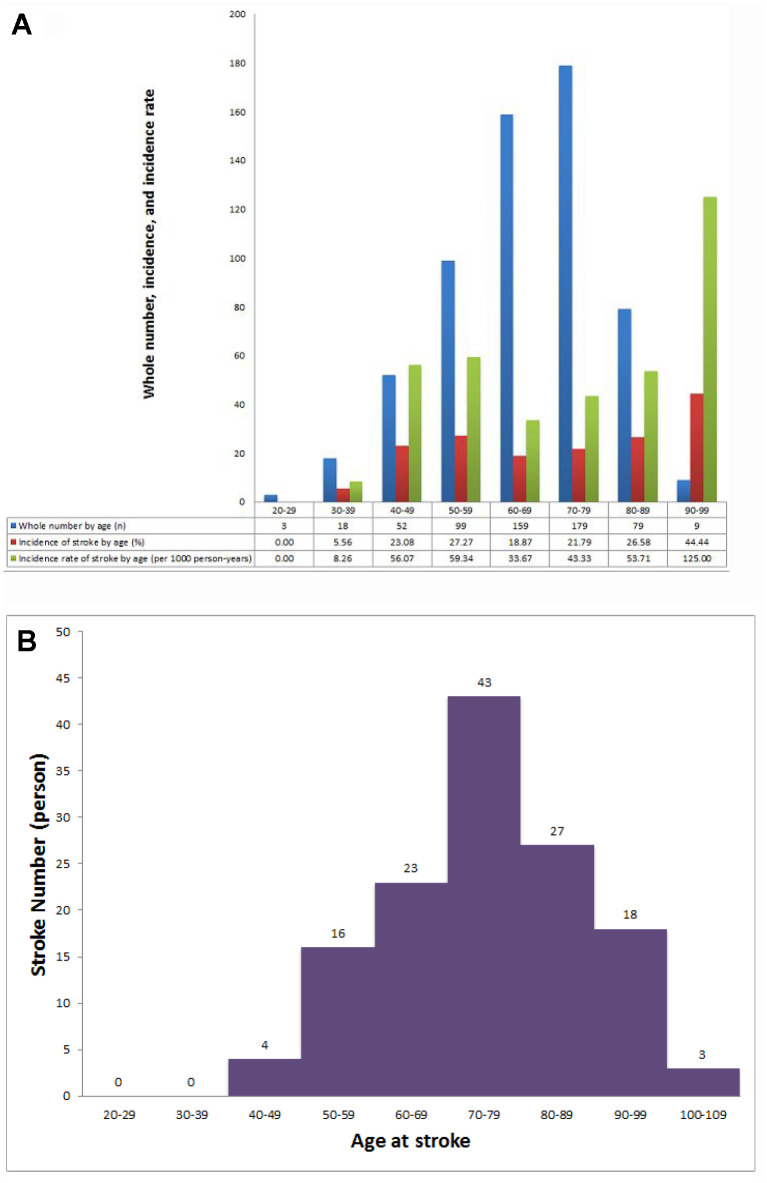
**Incidence of stroke in patients with HCM and AF.** (**A**) Whole number, stroke incidence, and incidence rate of stroke in patients with HCM and AF at different ages. (**B**) Distribution of age at stroke presentation.

**Figure 3:**
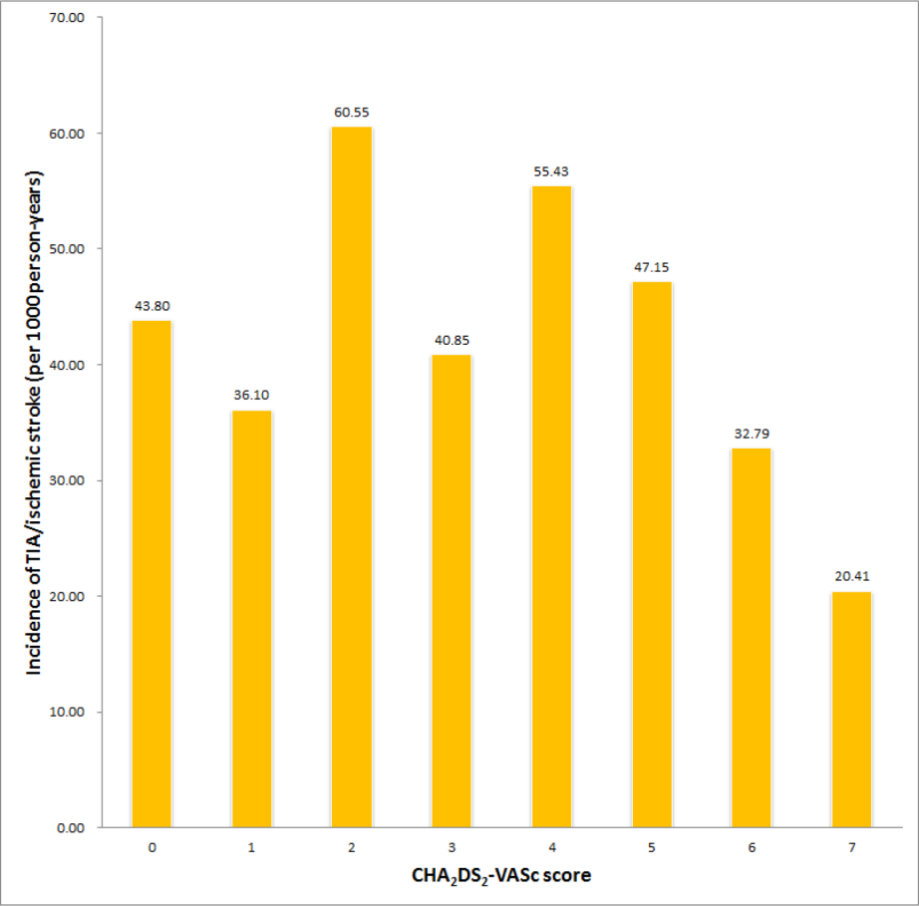
**Incidence of stroke in patients with HCM and AF with different CHA_2_DS_2_-VASc scores.**

In 18,724 subjects from the Taiwan National Health Insurance Research Database (NHIRD), 3970 subjects were excluded owing to use of an antiplatelet or an anticoagulant. A total of 598 patients (262 men, mean age 66.3±13.0 years) experienced new-onset AF during the follow-up period (Table 1). The enrollment of patients with HCM and AF is shown in Figure 1. The AF incidence in patients with HCM was 5.83 per 1000 person-years, and the overall incidence of AF-associated stroke was 24.14 per 1000 person-years. The median time and mean time from HCM to AF were 34.50 and 47.36 months, respectively. The median time and mean time from AF to transient ischemic attack (TIA)/ischemic stroke were 5.00 and 20.54 months, respectively. In the whole cohort, the cumulative incidence of TIA/ischemic stroke was 22.4%, and the incidence rate of TIA/ischemic stroke was 24.1 per 1000 person-years. Figure 2 demonstrates the incidence of TIA/ischemic stroke stratified by age. Figure 2A shows the age distribution of this HCM cohort with AF, the stroke incidence, and the incidence rate of TIA/ischemic stroke. The incidence rate of TIA/ischemic stroke increased from 8.26 to 56.07 per 1000 person-years between patients aged 30-39 years and those aged 40-49 years. In this cohort, the incidence of stroke by age was counted regardless of the age at stroke presentation (e.g., one male patient aged 36 years with a CHA_2_DS_2_-VASc score of 2 who experienced a stroke at age 47 years was counted in the 30-39 years age group). The age at stroke presentation is shown in Figure 2B. No occurrence of TIA/ischemic stroke was observed in HCM patients with AF aged <40 years. Figure 3 shows the incidence rate of TIA/ischemic stroke stratified by the CHA_2_DS_2_-VASc score. The incidence rate varied from 20.41 to 60.55 per 1000 person-years. The incidence rate of TIA/ischemic stroke was high even in patients with a low CHA_2_DS_2_-VASc score, with the highest incidence of 60.55 per 1000 person-years in patients with a CHA_2_DS_2_-VASc score of 2.

**Table 1:** Basic characteristics of 598 patients with atrial fibrillation.

Sex (male)	262 (43.8%)
Age (years)	66.3±13.0
Hypertension	347 (58.0%)
Type 2 DM	145 (24.2%)
Hyperlipidemia	132 (22.1%)
CKD	26 (4.3%)
PAOD	71 (11.9%)
CHF	223 (37.3%)
CAD	276 (46.2%)
Prior myocardial infarction	96 (16.1%)
Prior TIA/stroke	35 (5.9%)
CHA_2_DS_2_-VASc score	2.9±1.7
Medication	
Beta-blocker	196 (32.8%)
CCB	197 (32.9%)
ACEI/ARB	151 (25.3%)
Aldactone	22 (3.7%)
Diuretics	97 (16.2%)
Statin	72 (12.0%)
Median time from HCM to AF (months) (quartile)	34.50 (12.0, 74.25)
Mean time from HCM to AF (months) (min, max)	47.36 (0, 191)
Median time from HCM to TIA/ischemic stroke (years) (quartile)	4.00 (1.00, 8.00)
Mean time from HCM to TIA/ischemic stroke (years) (min, max)	4.75 (0, 15.00)
Median time from AF to TIA/ischemic stroke (months) (quartile)	5.00 (0, 33.50)
Mean time from AF to TIA/ischemic stroke (months) (min, max)	20.54 (0, 185)
Cumulative incidence of AF (%)	4.05
Incidence rate of AF (per 1000 person-years)	5.83
Cumulative incidence of TIA/ischemic stroke (%)	22.4
Incidence rate of TIA/ischemic stroke (per 1000 person-years)	24.1
Mortality (per 1000 person-years)	49.8

Abbreviations: DM, diabetes mellitus; CKD, chronic kidney disease; PAOD, peripheral arterial occlusive disease; CHF, congestive heart failure; CAD, coronary artery disease; TIA, transient ischemic attack; CCB, calcium channel blocker; ACEI/ARB, angiotensin-converting enzyme inhibitor/angiotensin receptor blocker

The predictors of TIA/ischemic stroke in patients with the AF are shown in Table 2. In univariate Cox proportional regression analysis, older age (continuous; hazard ratio 1.04, 95% confidence interval [CI] 1.02-1.06, p<0.001), prior TIA/ischemic stroke (hazard ratio 2.26, 95% CI 1.14-4.49, p=0.02), and CHA_2_DS_2_-VASc score (hazard ratio 1.18, 95% CI 1.06-1.32, p=0.003) were significantly associated with TIA/ischemic stroke. Multivariate analysis identified age (continuous; hazard ratio 1.04, 95% CI 1.02-1.06, p=0.001) and prior TIA/ischemic stroke (hazard ratio 2.82, 95% CI 1.27-6.25, p=0.011) to be significantly associated with TIA/ischemic stroke.

**Table 2:** Univariate and multivariate Cox regression analyses of transient ischemic attack/ischemic stroke in patients with atrial fibrillation.

	**Univariate analysis**		**Multivariate analysis**	
**Variables**	**HR (95% CI)**	**p**	**HR (95% CI)**	**p**
Sex (male)	0.78 (0.55-1.10)	0.153		
Age (continuous)	1.04 (1.02-1.06)	<0.001	1.04 (1.02-1.06)	0.001
Hypertension	1.08 (0.77-1.53)	0.656		
Type 2 DM	1.54 (0.94-2.52)	0.088		
Hyperlipidemia	0.91 (0.59-1.41)	0.666		
PAOD	0.87 (0.51-1.50)	0.626		
CHF	0.94 (0.67-1.33)	0.942		
CAD	0.92 (0.65-1.30)	0.632		
Prior myocardial infarction	1.38 (0.80-2.38)	0.248		
CKD	0.97 (0.35-2.65)	0.952		
Prior TIA/ischemic stroke	2.26 (1.14-4.49)	0.020	2.82 (1.27-6.25)	0.011
Beta-blocker	0.69 (0.48-1.00)	0.052		
CCB	0.85 (0.60-1.23)	0.391		
ACEI/ARB	0.85 (0.57-1.26)	0.404		
Aldactone	0.84 (0.11-5.83)	0.836		
Diuretics	0.98 (0.60-1.57)	0.921		
Statin	0.68 (0.38-1.21)	0.192		
CHA_2_DS_2_-VASc score	1.18 (1.06-1.32)	0.003	1.01 (0.88-1.17)	0.850

Abbreviations: HR, hazard ratio; CI, confidence interval; other abbreviations are as in Table 1.

## DISCUSSION

In this study, we investigated the risks of ischemic stroke in HCM patients with AF in a Taiwan population cohort with a 15-year follow-up period. We found that among the components of the CHA_2_DS_2_-VASc score, age is the main crucial predictor.

The pathophysiology of AF in HCM is complex. Patients with HCM have risks of hemodynamic decompensation and loss of the atrial contribution to left ventricular filling, which is the principal mechanism leading to AF or atrial arrhythmia. A thick myocardium with impaired diastolic relaxation leads to elevated left ventricular pressure, further elevates left atrial pressure, and induces atrial stress, forming an atrial substrate and finally resulting in the development of AF. Left atrial dilatation has been suggested to be a consequence of impaired diastolic function in patients with HCM. In some HCM patients with left ventricular outflow tract obstruction, mitral valve insufficiency further enhances the left atrial volume [1, 9]. The association between left atrial size and the presence of AF has been confirmed [10, 11]. One study has demonstrated that each 1 mm increase in the left atrial size was associated with a mild elevation of the ischemic stroke risk with a hazard ratio of just above 1 [12].

In our study, the AF incidence in patients with HCM was 5.83 per 1000 person-years, and the overall incidence of AF-associated stroke was 24.14 per 1000 person-years. Our previous study using the same cohort demonstrated that non-AF patients with HCM aged >65 years and with similar CHA_2_DS_2_-VASc scores had a significantly higher risk of ischemic stroke than AF patients without HCM [13]. Our finding further supports the notion that aging is an independent risk factor for ischemic stroke in patients with HCM.

In a prospective delayed-enhanced magnetic resonance imaging (MRI) study, a higher CHADS_2_ score was associated with an increased amount of left atrial fibrosis [14]. The study suggested that both HCM and a high CHA_2_DS_2_-VASc score could contribute to left atrial dysfunction. One recent retrospective study also showed that HCM with paroxysmal AF was associated with higher left atrial volume, lower left atrial ejection fraction, lower global peak longitudinal left atrial strain, and higher amount of left atrial late gadolinium enhancement, which are all hallmarks of global atrial cardiomyopathy [15]. AF is underrecognized among HCM patients. The diagnosis of AF in HCM patients may be difficult when the AF is paroxysmal or asymptomatic. In one study, AF had not been previously documented before stroke events in more than half of the patients with HCM [16]. The prevalence of AF in patients with HCM is high. A Holter study demonstrated that 3% of HCM patients had paroxysmal AF [17]. The prevalence of AF increased up to 25% when implanted devices were used to detect AF [18, 19]. Therefore, HCM patients with cryptogenic stroke should undergo careful and detailed AF screening with repetitive electrocardiographic monitoring or implantable loop recorders.

Interestingly, many studies found that patients with HCM have a high risk for embolic events, even those without AF or those with a zero CHA_2_DS_2_-VASc score, suggestive of HCM-related atrial myopathy [5, 6, 16]. Logically, patients with concomitant HCM and AF should have higher risks even with a zero CHA_2_DS_2_-VASc score. Accordingly, current guidelines recommend that all HCM patients with AF should receive oral anticoagulant treatment regardless of the CHA_2_DS_2_-VASc score [5, 20]. However, studies assessing thromboembolic rates according to the CHA_2_DS_2_-VASc score in HCM patients with AF yielded conflicting results. One study from China showed that a CHA_2_DS_2_-VASc score of ≤1 was associated with an annual thromboembolic incidence of only 0.9% in the first year and 0.5% per 100 patient-years at a median follow-up of 2.4 years [21]. Other studies have reported higher thromboembolic risks [2, 5, 6, 22]; for example, Jung et al. demonstrated that HCM patients with AF categorized as “low risk” based on the CHA_2_DS_2_-VASc score (i.e., score 0 in men or 1 in women) had an unadjusted rate of ischemic stroke and composite thromboembolism endpoint of 3.38% and 4.02% per year, respectively [22]. In the subgroup analysis of a Korean database of HCM patients, the incidence of AF-associated stroke was 1.49 per 100 person-years in patients aged <45 years and 1.48 per 100 person-years in patients with a low CHA_2_DS_2_-VASc score (0 or 1) [4]. Another study conducted in Italy also demonstrated a high annual stroke rate of 2.49% in HCM patients with AF [2]. In our study, the incidences of stroke were 43.80 and 36.10 per 1000 person-years in patients with a CHA_2_DS_2_-VASc score of 0 and 1 respectively, which are much higher than the results in Korea. The main reasons for the discrepancy are not clear but might be related to age at HCM diagnosis, racial differences, or differences in genetic background. Notably, in our cohort, no TIA/ischemic stroke events occurred in patients aged <40 years. Moreover, in the 30-40 age group, only one patient experienced TIA/ischemic stroke after about 10 years, and the initial CHA_2_DS_2_-VASc score at index time in this patient was 2. Whether anticoagulant treatment should be used in younger (age <40 years) patients with AF needs further studies. Although younger HCM patients with AF may have less need for anticoagulant use, our results suggest that the risk of TIA/ischemic stroke should be reexamined every year, and an anticoagulant therapy should be started when the patients become older, regardless of the CHA_2_DS_2_-VASc score.

### Study limitations

This study had several limitations. First, because this study was based on a claims database, the estimation of the incidence of comorbidities, TIA, and ischemic stroke were based on physician reports. Second, the database does not provide several important clinical information such as left atrial size, left ventricular wall thickness, degree of diastolic dysfunction, and left ventricular ejection fraction, or laboratory data such as N-terminal pro-brain-type natriuretic peptide levels, which represent atrial stretch-related stress.

## CONCLUSION

In a Taiwanese cohort with concomitant HCM and AF, the overall incidence of stroke was low in patients with younger age and low CHA_2_DS_2_-VASc score. Age is an important predictor of TIA/ischemic stroke, and anticoagulation should be applied in older patients.

## MATERIALS AND METHODS

### Ethics statement

Investigation has been conducted in accordance with the ethical standards and according to the Declaration of Helsinki and according to national and international guidelines and has been approved by the authors’ institutional review board.

### Data source

This longitudinal cohort study is based on the NHIRD of Taiwan. The National Health Insurance program is managed by the Taiwanese government, with the majority (98%) of the Taiwanese population as mandatory subscribers. The NHIRD integrates medical and pharmaceutical information, including patients’ sociodemographic information, inpatient and outpatient services, pharmacy dispensing claims, procedures, and mortality data, with the International Classification of Diseases Ninth Revision Clinical Modification (ICD-9-CM) codes. The databases are open to researchers whose study protocols were approved by the official review committee. The protocol for this study was approved by the Institutional Review Board of National Taiwan University Hospital, which waived the requirement for informed consent.

### Study population

A total of 18,724 patients with prevalent HCM who were aged 18 years or older were identified from January 1997 to December 2013 from a systematic sample of 1,000,000 people from the NHIRD database, with a median follow-up period of 7 years. The diagnosis of HCM was made if a wall thickness of ≥15 mm in one or more left ventricular myocardial segments was detected by any imaging technique (echocardiography, cardiac MRI, or computed tomography) and could not be entirely explained by loading conditions. The index date for this study was the date of the first-time diagnosis of HCM (ICD-9-CM code 425.1). An AF diagnosis was identified as ICD-9-CM code 427.3. Patients were excluded if AF was diagnosed before the index date, if the AF was associated with valvular conditions such as mitral valve stenosis or prosthetic valve replacement, or if AF was not diagnosed. Patients were also excluded if they received antiplatelet or anticoagulation treatment. Comorbidities were recorded at the time of hospital discharge diagnosis or in the outpatient clinic according to the following ICD-9-CM codes: hypertension (401.X-405.X), diabetes mellitus (250.X, 249.X), hyperlipidemia (272.X), coronary artery disease (411.X-414.X, V17.3, V81.0), hospitalization for heart failure (428.0-428.3, 428.9), and chronic kidney disease (585.X-588.X; defined as glomerular filtration rate <60 mL/min/1.73 m^2^). The dispersion time and index time of medications, including angiotensin-converting enzyme inhibitors, angiotensin receptor blockers, beta-blockers, calcium channel blockers, and statins, were identified. The outcome was TIA (435.X) or ischemic stroke (434.X) in any ambulatory visit and discharge diagnosis.

### Statistical analysis

Continuous variables are shown as mean±standard deviation, whereas categorical variables are expressed as frequencies. Comparisons between groups were examined using Student’s t test for continuous variables and the χ2 test for categorical variables. The annual incidence, cumulative incidence, and incidence rate of stroke in the whole cohort and in subgroups by ages and CHA_2_DS_2_-VASc scores are depicted as histograms. Univariate and multivariate Cox proportional hazard regression analyses were used to adjust for potential confounders. Confounders were entered into multivariate regression analysis only if they showed a significant association with TIA or ischemic stroke in univariate regression analysis. All analyses were performed using SPSS version 25.0 (SPSS Inc., Chicago, IL, USA). Values of two-tailed p<0.05 were considered statistically significant.
